# Thin Films of Polyaniline-Based Nanocomposites with CeO_2_ and WO_3_ Metal Oxides Applied to the Impedimetric and Capacitive Transducer Stages in Chemical Sensors

**DOI:** 10.3390/polym15030578

**Published:** 2023-01-22

**Authors:** Beatriz Cotting Rossignatti, Amanda Portes Vieira, Martin Schwellberger Barbosa, Luís Miguel Gomes Abegão, Hugo José Nogueira Pedroza Dias Mello

**Affiliations:** 1 Physics Institute, Goiás Federal University, Samambaia Campus, Goiânia 74001-970, GO, Brazil; 2 Chemistry Institute, Goiás Federal University, Samambaia Campus, Goiânia 74001-970, GO, Brazil

**Keywords:** conducting polymer, metal oxide, nanocomposites, polyaniline, sensor, pH detection, electrochemistry

## Abstract

There is a recognized need for the development of cost-effective, stable, fast, and optimized novel materials for technological applications. Substantial research has been undertaken on the role of polymeric nanocomposites in sensing applications. However, the use of PANI-based nanocomposites in impedimetric and capacitive electrochemical sensors has yet to be understood. The present study aimed to explore the relationship between the sensitivity and linearity of electrochemical pH sensors and the composition of nanocomposites. Thin films of PANI/CeO_2_ and PANI/WO_3_ were deposited via spin coating for characterization and application during the electrochemical impedance and capacitance spectroscopy (EIS and ECS) transduction stages. The findings showed that the optimized performance of the devices was extended not only to the sensitivity but also to the linearity. An increase of 213% in the ECS sensitivity of the PANI/CeO_2_ compared to the metal oxide and an increase of 64% in the ECS linearity of the PANI/WO_3_ compared to the polymeric sensitivity were reported. This study identified the structure–property relationship of nanocomposite thin films of PANI with metal oxides for use in electrochemical sensors. The developed materials could be applied in devices to be used in different fields, such as food, environment, and biomedical monitoring.

## 1. Introduction

Conducting polymers (CP) represent a class of polymers that have gained attention due to the work of Shirakawa, MacDiarmid, and Heeger [1]. CP have a conjugated structure due to sp^2^ hybridized carbons in their backbone, which leads to the delocalization of π-electrons, ensuring their unique properties, with particular attention being paid to the electrical one [2]. The doping of the conjugated structure can improve the electrical conductivity and is responsible for the wide range of applications of CP [3]. There are several CP, such as polyacetylene (PA), polyaniline (PANI), polypyrrole (PPY), poly(p-phenylene) (PPP), poly(p-phenylenevinylene) (PPV), polythiophenes (PTH), and their derivatives. Among them, PANI is one of the most studied and applied CP due to its low cost, ease of synthesis, processability, deposition as a thin film, environmental stability, and possibility of forming hybrid structures [4,5,6].

The ability to tailor the mechanical, optical, and electrical properties of PANI renders the polymer a viable material for many applications, such as conductive adhesives, diodes, organic transistors, solar cells, capacitors, energy storage devices, ion-selective electrodes, chemical sensors, gas sensors, and biosensors [7,8,9,10,11,12,13,14,15]. PANI plays a central role in the development of sensors. Its ability to change its properties upon exposure to acids and bases, as well as some vapors and liquids, makes it a candidate for such applications [16]. PANI-based sensors and biosensors involve potentiometric, amperometric, conductometric, impedimetric, capacitive, and optical transducer stages [17,18,19,20]. The use of PANI-based thin films for pH sensors has been shown for different transduction systems, sample preparations, and applications. Mello and co-workers [21] and Chinnathambi and co-workers [22] showed optical pH sensors with electrodeposited PANI samples. Mello and co-workers [23] and Vieira and co-workers [24] showed potentiometric PANI-based pH sensors with FET platforms. Despite the polymer’s many qualities, PANI-based chemical sensors may suffer from sensitivity, linearity, selectivity, or stability limitations. Such limitations might be overcome by introducing a secondary material into the PANI, thereby forming a polymeric composite [25]. Regarding the use of PANI-based composites, Nguyen and co-workers described a conductometric platform fabricated via microlithography using a PANI and poly(vinyl butyral) (PVB) blend layer as a pH sensor for water quality monitoring [26].

Polymeric composite synthesis, fabrication, characterization, and application are significant areas of interest within the materials science field due to the potential to eliminate the limitations of polymers in different application fields [27,28,29,30]. Combining PANI with a secondary nanocomponent, such as metallic nanoparticles, metal oxide nanoparticles, carbon compounds, or polymers, enhances its functionalities and performance, offering an efficient design [31]. To date, several studies have demonstrated that synergic interactions expand the scope and application of PANI-based nanocomposites by improving their known properties and promoting their novel features [32].

Nanocomposites of PANI with metal oxides have enhanced properties due to the synergistic interactions between the constituents that are useful for applications, such as sensors and biosensors, photovoltaics, and batteries [25,33]. Moreover, several studies have used nanocomposites of PANI with metal oxides for sensing applications [34]. One reason for the enhanced sensor response of PANI with metal oxide nanocomposites is the development of electron-conducting pathways in the material, which improve the device’s efficiency [27]. To date, different authors have investigated the application of nanocomposites in sensors, such as PANI/TiO2 (titanium dioxide) [35], PANI/WO3 (tungsten trioxide) [36], and PANI/CeO2 (cerium dioxide) [37]. While the first two were prepared by physically mixing the constituents, PANI, and metal oxide nanoparticles in solutions for deposition, the last one was prepared via the in situ self-assembly of the composite prior to deposition. As sensors, the nanocomposites presented optimized performance compared to their constituents. Previous studies have shown that electrochemical sensors and biosensors based on PANI nanocomposites are promising devices. Still, drawbacks must be overcome, such as the need for cost-effective, fast, and reliable analysis methods to achieve a dedicated sensing device using PANI-based composites [38]. Such is the case for pH sensors, which have importance in the quantification of chemicals and bio-chemical analytes in environmental and biomedical monitoring.

Recently, we demonstrated that pristine PANI can be used as a promising platform for chemical sensors based on the use of the electrochemical impedance spectroscopy (EIS) and EIS-based electrochemical capacitance spectroscopy (ECS) transducer stages [39]. The use of the capacitive properties of PANI in chemical sensors has been novel to date. Our previous work showed that the oxidation state of the PANI thin film directly influences its response in each setup. Therefore, this work reveals how to achieve a low-cost and effective electrochemical pH sensor using PANI-based metal oxide nanocomposites, particularly PANI/CeO_2_ and PANI/WO_3_, in the form of thin films to further explore and improve the previous approach. The thin film production was accomplished using the well-known spin-coating technique, which is an inexpensive, straightforward, and fast method for producing thin films. The analysis methods were based on EIS and ECS. Such methods use a proper electric equivalent circuit model (EECM) to interpret the impedance and capacitance changes in the electrode/electrolyte interface [40,41,42,43]. The equivalent circuit used in this work is widely applied to study polymer-coated metal systems [44,45] due to allowing the decoupling of the electron transfer from the mass-transport process [46]. The performance of the sensor with nanocomposites is compared to that of its constituents regarding the sensitivity and linearity of the devices. We show that the synergic interactions between the constituents influence the structure–property relationship of the composites, with a significant effect on the sensor’s figures of merit. 

## 2. Materials and Methods

### 2.1. Materials

The fluorine-doped tin oxide (FTO) thin film deposited over a glass substrate (7 Ω/sq.), PANI (PANI-EB, M_W_ 50,000 g/mol), hydrous dibasic sodium phosphate (99%), sodium tungstate (Na_2_WO_4_.2H_2_O, 99%), oxalic acid (H_2_C_2_O_4_.2H_2_O, 99%), potassium sulfate (K_2_SO_4_, 99%), cerium nitrate (CeNO_3_.6H_2_O, 98%), and N-Methyl-2-pyrrolidone (NMP, 99%) were purchased from Sigma-Aldrich. The citric acid (99.5%) was obtained from Vetec, Brazil. Glass slides (KB7) were used as the substrate for the sample deposition used in the characterization. All the chemicals were used as received without any further purification.

### 2.2. CeO_2_ and WO_3_ Synthesis

The synthesis of the cerium dioxide nanorods (CeO_2_) via the hydrothermal route followed the procedures described by Mai and co-workers [47]. First, 0.434 g of cerium nitrate was dissolved in 5 mL of deionized (DI) water, before 15 mL of NaOH 9 mol/L was added to the solution under vigorous stirring. Then, the solution was transferred to a sealed polytetrafluoroethylene autoclave and heated in a conventional oven at 100 °C for 24 h. The resultant white precipitate was washed and centrifuged with water and ethanol three times and dried at 80 °C for 24 h, yielding a yellow powder.

The synthesis of the hexagonal phase tungsten oxide (h-WO_3_·H_2_O) microplates via the hydrothermal route followed a procedure described by Gu and co-workers [48]. Briefly, 0.73 g of sodium tungstate was dissolved in 20 mL of DI water, followed by the addition of 0.71 g of oxalic acid under stirring. Hydrochloric acid 3 mol/L was added dropwise to set the pH to 2, followed by the addition of 0.16 g of potassium sulfate. The solution was transferred to a sealed polytetrafluoroethylene autoclave and heated in a conventional oven at 160 °C for 12 h. The resultant bright yellow powder was washed and centrifuged with water and ethanol three times and dried at 80 °C for 24 h.

### 2.3. Sample Preparation and Characterization

The PANI, metal oxide (CeO_2_ and WO_3_), and nanocomposite (PANI/CeO_2_ and PANI/WO_3_) thin films were deposited via spin coating (1000 rpm, for 60 s) using a G3P-8 spin coater (SCS). For sensing and characterization purposes, FTO and glass thin films were used as substrates after cleaning via ultrasonication (DI water, ethanol, and acetone, 10 min each). The PANI was deposited from a weight ratio of 1:400 polymer:solvent (NMP) solution. The CeO_2_ and WO_3_ were deposited from a 60 and 11 mg/mL dispersion in NMP, respectively. The PANI solution and metal oxide dispersions were stirred and ultrasonicated for 1 h each before use. The nanocomposite films were deposited from a 50:50 (*V*/*V*) solution prepared from the precursor solution and dispersions. After mixing, the composite solutions were stirred and ultrasonicated for 1 h each before use. The samples were annealed on a hot plate for 20 min at 120 °C.

Scanning electron microscopy (SEM) and energy-dispersive X-ray spectroscopy (EDS) characterizations were performed in the samples deposited over glass substrates using a JEOL JSM—6610 system with an operation voltage of 15–20 kV. Prior to the analysis, a 5 nm Au layer was sputtered over the samples to enhance the electrical conductivity. The UV-VIS absorbance spectra of the samples were recorded using a Lambda 1050 WB spectrophotometer (PerkinElmer, Waltham, MA, USA). The selected absorbance spectral window ranged from 350 to 850 nm with a 0.5 nm step. The samples’ profiles for thickness and average surface roughness determination purposes were obtained using a DektakXT stylus profilometer (Bruker, Billerica, MA, USA). The recorded data were analyzed using the Mountains Map software (Digital Surf, Besançon, France). The results were analyzed for the absorbance above 370 nm to minimize interference by the KB7 glass substrate. All the experiments were performed at room temperature, i.e., 298 K.

### 2.4. Sensor Measurement

An AUTOLAB potentiostat (Metrohm, Herisau, Switzerland) with an FRA module controlled by the NOVA software was used for the electrochemical measurements. A conventional three-electrode electrochemical cell system was used. The samples (PANI, CeO_2_, WO_3_, PANI/CeO_2_, and PANI/WO_3_ thin films) were used as working electrodes. The reference electrode was an Ag|AgCl (3 mol/L) electrode, and platinum foil was used as the counter electrode. All the experiments were performed at room temperature. The cyclic voltammetry (CV) was performed with a scan rate of 100 mV/s in a scan window between—0.2 and 1.2 V vs. Ag|AgCl. The electrochemical impedance spectroscopy (EIS) measurements were performed under an open circuit potential (OCP) vs. Ag|AgCl with an AC frequency ranging from 100 mHz to 100 kHz. The AC voltage amplitude used was 10 mV RMS. The films were carefully washed in distilled water between the measurements. The EIS-derived electrochemical capacitance spectroscopy (ECS) analyses consisted of converting the complex impedance into complex capacitance, as discussed elsewhere [49].

McIlvaine buffer solutions, ranging from a pH of 2 to 8, were used as electrolytes to obtain the calibration curve of the pH sensor. The relative response at any specific pH and frequency was defined as $RR_{pH}^{\omega }\left(\%\right)=\left(RR_{pH}^{\omega }/RR_{Max}\right)\times 100$, where $RR_{pH}^{\omega }$ is the electrochemical capacitance ($C_{\bar{\mu }}$) or the charge-transfer resistance ($R_{ct}$) for the ECS and EIS systems, respectively, and $RR_{Max}$ stands for the maximum response for the specific sample. The sensor’s linearity was obtained from the coefficient of determination (R^2^) of the fitted calibration curves. The linearity parameter L was calculated as R^2^ times 100. All the experiments were performed in triplicate, and the results are presented as a function of the mean and standard deviation values.

## 3. Results

**Figure 1:**
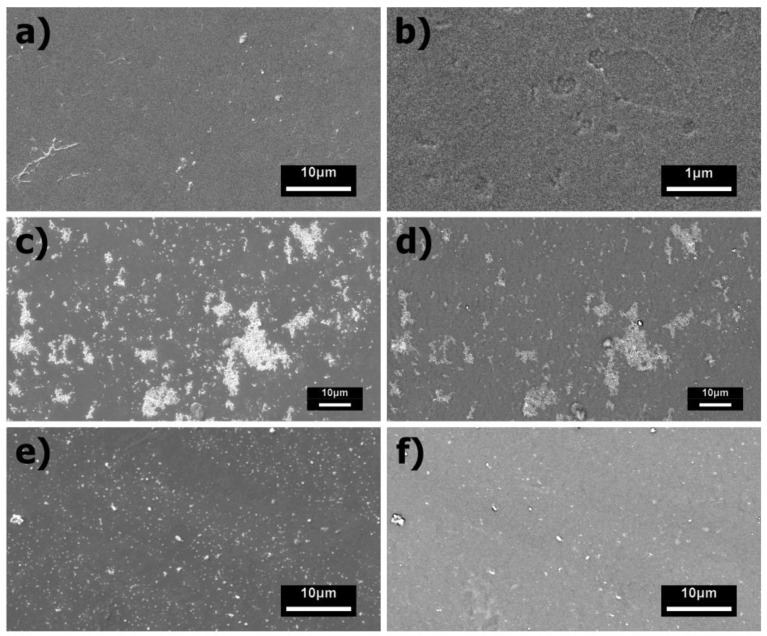
SEM characterization of the pristine PANI and composite films deposited over glass substrates when using the secondary electrons (SE) as signals for high-resolution images or backscattered electrons (BSE) to enhance the atomic number contrast between the PANI and oxide phases in the composite films. SE images of the pristine PANI films are presented in (**a**,**b**). SE and BSE images of the PANI/CeO_2_ films are presented in (**c**,**d**), respectively. SE and BSE images of the PANI/WO_3_ films are presented in (**e**,**f**), respectively.

The surface morphology and multi-phase elemental composition of the PANI, PANI/CeO_2_, and PANI/WO_3_ films deposited over glass substrates were investigated by means of SEM-EDS (Figure 1). Two different operation modes were used for the collection of the images: secondary electrons (SE) for images with higher spatial resolution and backscattered electrons (BSE) for images with spatially resolved atomic number contrast (Z-contrast). The pristine PANI films exhibited smooth continuous morphology (Figure 1a,b). The PANI/CeO_2_ exhibited a secondary phase composed of several clusters of nanostructures spread across the surface (Figure 1c), which also showed a high atomic number contrast in the BSE images (Figure 1d) due to the high atomic number of Ce (Z = 52). Likewise, the PANI/WO_3_ films presented a secondary phase composed of dispersed plate-like particles with sizes ranging from a few hundred nanometers to a few micrometers (Figure 1d), with a high atomic number contrast in the BSE images (Figure 1e) due to the presence of W (Z = 74). For both films, the EDS analysis confirmed the presence of the respective elements in the secondary phases (Figure S1). Together, these results indicate that the composite phases were successfully prepared via the co-dispersion spin-coating methodology, with the incorporation of the respective oxide materials into the surface of the polymer film.

**Figure 2:**
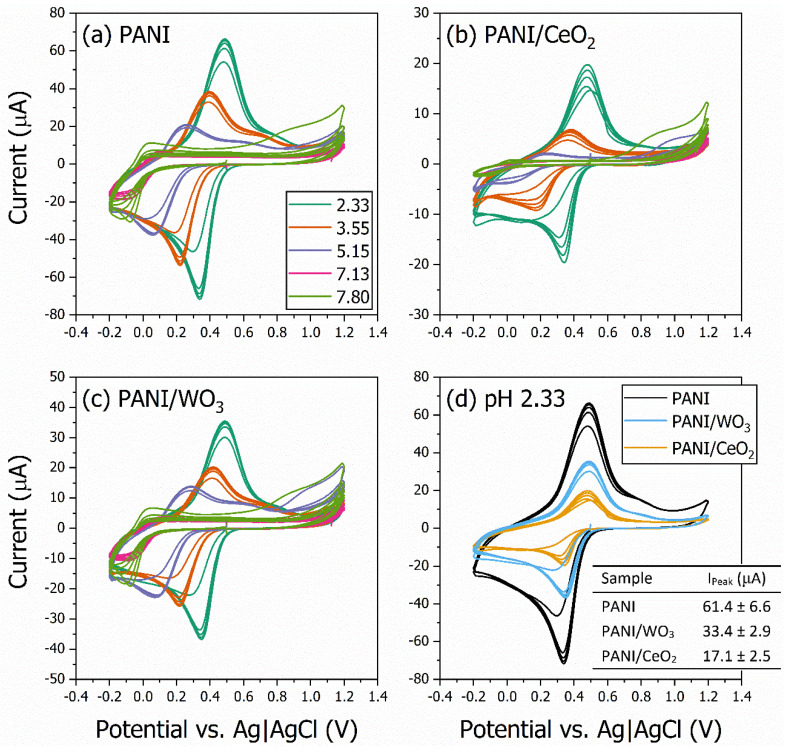
Cyclic voltammetry findings collected in different pH buffer electrolytes used as working electrode FTO substrates modified with: (**a**) PANI, (**b**) PANI/CeO_2_, and (**c**) PANI/WO_3_ nanocomposites. The reversible redox peaks of PANI-based materials are present. The peak current is proportional to the buffer solution pH. In (**d**), the cyclic voltammograms of the three samples are compared for the same buffer solution with a pH of 2.33. The inset shows the table with the peak currents. The I_Peak_ is influenced by the composite composition.

The cyclic voltammograms (CVs) of the pH sensors for the different materials are given in Figure 2. Figure 2a–c show the CVs for the PANI, PANI/CeO_2_, and PANI/WO_3_ films, respectively. Figure 2d compares the CVs of the three samples for the same buffer solution with a pH of 2.33. It is possible to observe the characteristic reversible redox peaks of PANI-based materials in the CVs. The films made of the metal oxides did not present reversible redox peaks (see Figure S2 in the SI). From the graph, one can also see that the oxidation and reduction potentials are different for each pH. This may be attributed to the electrostatic interaction of the ions in the electrolyte with the chemical groups in the polymer [50]. It is apparent from the figure that, for each sample, the oxidation and reduction peak current values correlated with the pH of the buffer solution and were different among the samples for the same pH.

The reduction/oxidation reactions and doping/dedoping process occurring on the film surface depend on the pH due to the variation in the ion concentration. PANI-based materials are protonated in their quinoid rings [51], and this process is responsible for the ionic current measured via CV. Therefore, increasing the pH will cause a decrease in the peak current measured. The differences in the CVs among the samples can be explained in part by the fact that the reactions occurring on the surface of the films are dependent on the composition of the nanocomposite.

It is also possible to observe that the materials presented different values for the peak current at each pH, with the composite materials presenting a smaller response (Figure 2d). For the buffer solution with a pH of 2.33, the PANI sample presented a higher current, followed by the PANI/WO_3_ composite and then the PANI/CeO_2_ film. The peak currents (I_Peak_) were 61.4 ± 6.6, 33.4 ± 2.9, and 17.1 ± 2.5 μA, respectively (as shown in the inset in Figure 2d). A decrease in the peak current is expected for both composites due to the absence of the voltametric response of the individual oxides (see Figure S2 in the SI).

**Figure 3:**
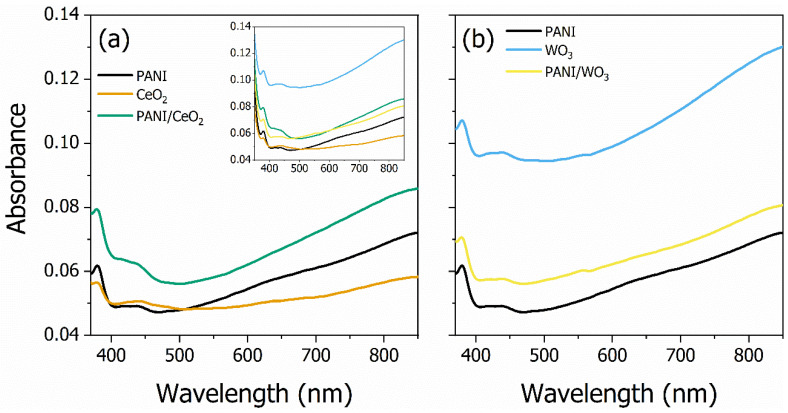
UV-VIS absorption spectra of all the thin films investigated in this work. The CeO_2_ group (PANI, CeO_2_, and PANI/CeO_2_ thin films) and WO_3_ group (PANI, WO_3_, and PANI/WO_3_ thin films) absorbance spectra are presented in (**a**,**b**), respectively. The (**a**) inset shows the entire spectra of PANI and its composites. The thin films’ absorbance changes with the presence of the metal oxide in the PANI matrix.

The results, as shown in Figure 2d, indicate that the electrochemical response of the nanocomposite films depends on the metal oxide. The nanocomposite samples were prepared in a 50:50 ratio of polymer and metal oxide, and the inset table in Figure 2d shows that the I_Peak_ of the PANI/WO_3_ is about 54% of the PANI I_Peak_. By contrast, the I_Peak_ of the PANI/CeO_2_ is about 27% of the PANI I_Peak_, suggesting that the decrease in the I_Peak_ of the PANI/WO_3_ to approximately half of the PANI value is a result of the film composition. The same would be expected for the PANI/CeO_2_ film. However, a larger decrease in the I_Peak_ for the PANI/CeO_2_ is observed than for the PANI/WO_3_. A possible explanation for this might be that the CeO_2_ interacts with the PANI quinoid rings, decreasing the overall protonation potential of the composite [52]. These results agree with those obtained via UV-VIS spectroscopy of PANI thin films. The PANI/CeO_2_ film presented a typical protonated PANI (PANI-ES) peak [53,54] (see Figure S3 in the SI) characterized by a band at 440 nm (Figure 3a).

The experimental UV-VIS absorption spectroscopy findings are shown in Figure 3. Before discussing the samples’ absorbance behavior, it is essential to point out that for the spectral region above 650 nm, the absorbance increases due to light scattering, causing transmittance variation, which should not be considered a pure absorbance effect. With that in mind, and emphasizing that a KB7 glass is the samples’ substrate, one could infer that the existent peaks *c.a.* at 390 nm are due to the substrate. Two spectral regions of interest are around 440 nm and 620 nm. The lowest energy region (around 620 nm) is where the maximum absorbance of PANI exists [53,54]. The highest energy region (around 440 nm) could be related to the interaction of CeO_2_ and WO_3_ with the PANI quinoid rings, which is responsible for the existent absorption peaks and the voltammogram behavior, as previously discussed.

The UV-VIS spectra of the CeO_2_ group (PANI, CeO_2_, and PANI/CeO_2_ thin films) are shown in Figure 3a. The CeO_2_ thin film (solid orange line) does not present the same low energy band as the PANI thin film (solid black line), in which the characteristic absorbance band is revealed by the discrete shoulder centered *c.a.* at 620 nm, which is almost masked by the scattering light effect. Regarding the high-energy band, centered *c.a.* at 440 nm, the PANI/CeO_2_ nanocomposite absorbance spectrum increases, probably due to the interaction of CeO2 with the PANI quinoid rings, as mentioned before. Regardless of the spectral bands’ nature, there is an overall increase in the absorbance of the PANI/CeO_2_ nanocomposite, as expected once the nanocomposite has more material adsorbed in the substrate. Moreover, the metal oxide in the polymeric matrix further contributes to the higher density of light scatterers, as also revealed in the absorption spectrum of the nanocomposite, i.e., the nanocomposite’s absorbance (solid green line) has a higher scattering of light above 650 nm.

Figure 3b shows the UV-VIS spectra of the WO_3_ group (PANI, WO_3_, and PANI/WO_3_ thin films). The WO_3_ thin film presents a higher absorbance (solid cyan line), with a broad band at low energy in the visible spectra. The PANI/WO_3_ nanocomposite absorbance (solid yellow line) has a higher value than the PANI film (solid black line), which might be due to the contributions of three factors: the increase in the scattering centers, the polymer chain, and oxide nanoparticles in the film, as observed for the PANI/CeO_2_. Further data analysis reveals that the PANI/WO_3_ and PANI present similar spectral features, suggesting a negligible interaction between the WO_3_ and PANI chemical groups.

**Figure 4:**
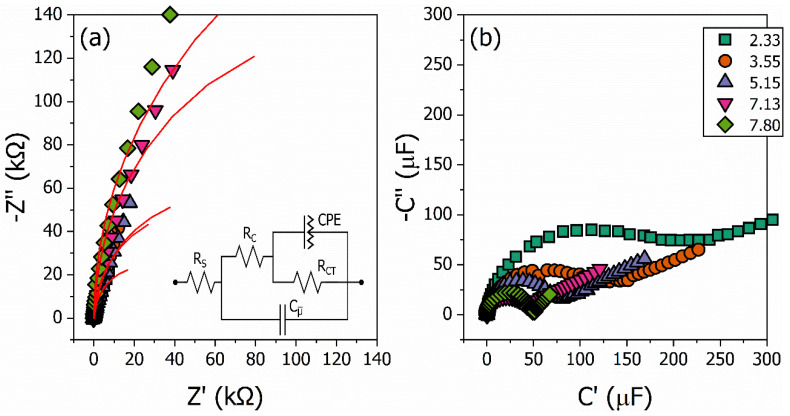
Impedimetric Nyquist diagram of the complex impedance (**a**) and capacitive correlated Nyquist diagram, i.e., the capacitive spectra plot (**b**) for the PANI/WO_3_ nanocomposite. The impedimetric graph is used to obtain the charge-transfer resistance, $R_{CT}$, for each buffer solution from the fitting of an equivalent circuit model (illustrated in the inset), while the capacitance graph is used to obtain the electrochemical capacitance, $C_{\bar{\mu }}\left(\omega \right)$, of the system from a phase-frequency analysis. The red lines are the adjusted curves for the equivalent circuit model.

We performed the pH sensor measurements using EIS and ECS as transducing platforms following our previously established methodology, again using pristine PANI, metal oxide films, and PANI–oxide composites. The results for the PANI/WO_3_ nanocomposite material are shown in Figure 4. The EIS and ECS spectra are acquired for all the samples for each pH buffer solution. A constant DC voltage perturbated by a small oscillating amplitude is applied to the system during the measurements. The reduction and oxidation reactions and the protonation/deprotonation process cause variations in the interfacial impedance and capacitance of the samples under different buffer solutions. The changes in these properties can be measured and estimated. The PANI/WO_3_ Nyquist diagram of complex impedance is shown in Figure 4a (data for the other samples are not shown here). The $R_{CT}$ changes are provided by the electric equivalent circuit model (EECM), which is applied to the Nyquist diagram. In the same way, the capacitive spectra plots are shown in Figure 4b. The ECS spectra are based on the mathematical conversion of the complex impedance into a complex capacitive signal, i.e., $C^{*}\left(\omega \right)=1/i\omega Z^{*}\left(\omega \right)$. The electrochemical capacitance parameter of the circuit, $C_{\bar{\mu }}\left(\omega \right)$, is obtained from the phase-frequency analysis.

The EIS spectra present a partially shown semicircle profile in the kΩ range. Each pH is associated with an interfacial charge-transfer resistance, $R_{ct}$, which is used as a transducing signal for resistive sensing. This pattern of the EIS spectra is in keeping with previous observational studies [55], which associate electrodes not only with the redox probe attached to it but also need to account for the electrochemical capacitance, $C_{\bar{\mu }}\left(\omega \right)$, associated with a redox reaction, thereby allowing for capacitive transduced sensing. These experimental results could be related to the nature of the polymer, because the redox probe is the PANI structure itself, meaning it is capable of undergoing redox processes.

The EECM is shown in the inset in Figure 4a. The equivalent circuit for polymer-coated electrodes is composed of the electrolyte solution resistance, $R_{S}$, the polymeric pore resistance for electrolyte penetration, $R_{C}$, the charge-transfer resistance, $R_{CT}$, the constant phase element, CPE [56], representing the charge accumulation at the interface, which is classically interpreted as the electric double layer capacitance, $C_{DL}$, and the electrochemical capacitance, $C_{\bar{\mu }}$, which is classically presented as the electrostatic coating capacitance, $C_{C}=\epsilon \epsilon_{0}A/d$, and updated to the electrochemical DOS occupancy interpretation [57]. The decrease in the buffer solution’s pH (acidic pHs) contributes to the increase in the redox activity and protonation of PANI-based materials. A consequence of this is a decrease in the impedance of the system. This impedance variation can be monitored using the $R_{CT}$ variations.

**Figure 5:**
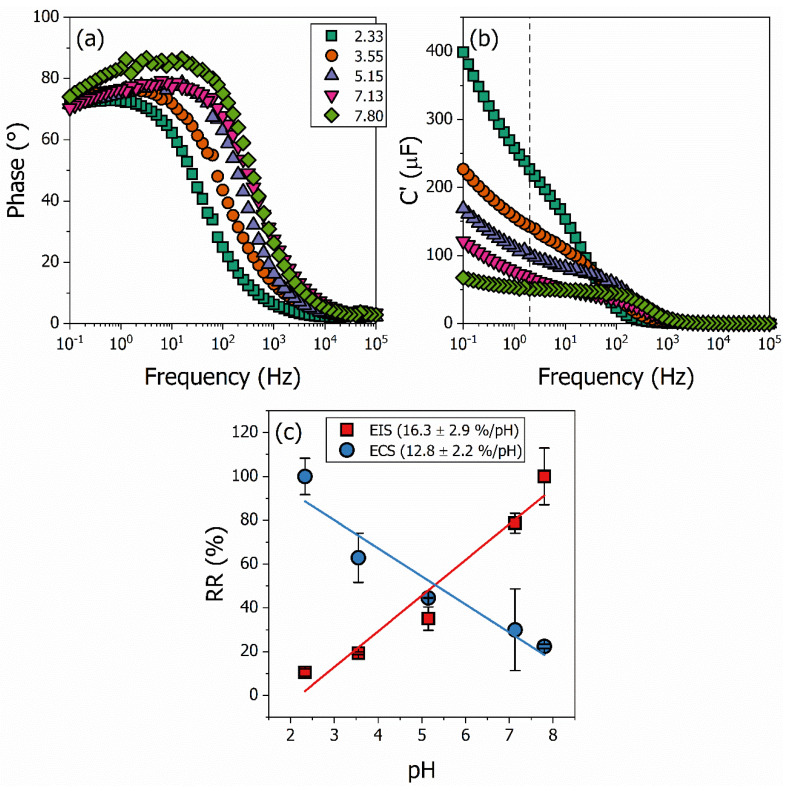
Electrochemical capacitance spectroscopy measurement analysis. The EIS phase angle vs. frequency in (**a**) and the capacitance data for the PANI/WO_3_ nanocomposite for each pH buffer solution in (**b**). From the frequency of the maximum phase, 1.99 Hz, the relative response of the capacitance is obtained for each pH buffer solution. Calibration curves using the relative response of the electrochemical capacitance, $C_{\bar{\mu }}$, and the charge-transfer resistance, $R_{CT}$, are plotted as a function of the pH in (**c**).

The ECS measurements of the PANI/WO_3_ nanocomposite thin film are shown in Figure 5. The analysis procedure was previously described [39] and is accurate for obtaining electrochemical information using EIS and EIS-derived capacitive methods for polymeric-based materials. The analysis of the ECS data involves determining the frequency of the maximum phase angle from the phase angle-frequency graph (Figure 5a) for the more acidic pH buffer (2.33). After determining the frequency, the real capacitance (C′) at the specific frequency is obtained from the real capacitance frequency graph (Figure 5b). For the PANI/WO_3_ composite, the maximum phase angle is obtained at 1.99 Hz, as depicted by the dashed line in Figure 5b.

The relative responses of the electrochemical capacitance, $C_{\bar{\mu }}$, and the charge-transfer resistance, $R_{CT}$, are plotted as a function of the pH in Figure 5c. The $C_{\bar{\mu }}$ is represented by the blue circle and the $R_{CT}$ by the red square. The EIS and ECS relative response variations are caused by the surface charge density and electric field distribution around the film/solution interface changing with the buffer solution [58]. The relative capacitance decreases linearly with an increasing pH in the range from 2 to 8. This can be attributed to the changes in its electronic density, mainly its redox state, that contribute to the $C_{\bar{\mu }}$, which are caused by the chemical reaction (reduction/oxidation and protonation/deprotonation) of the composite film. The structural [59] and electrostatic changes [60] are not the only ones responsible for the capacitance changes in the samples. A sensitivity of 12.8 ± 2.2%/pH and a linearity of 89% are obtained. The relative variation of the $R_{CT}$ increases with the increasing pH in the range from 2 to 8, and as for the capacitance, is attributed to the chemical reactions of the composite film. An EIS-based sensitivity of 16.3 ± 2.9%/pH and linearity of 92.8% are obtained.

**Figure 6:**
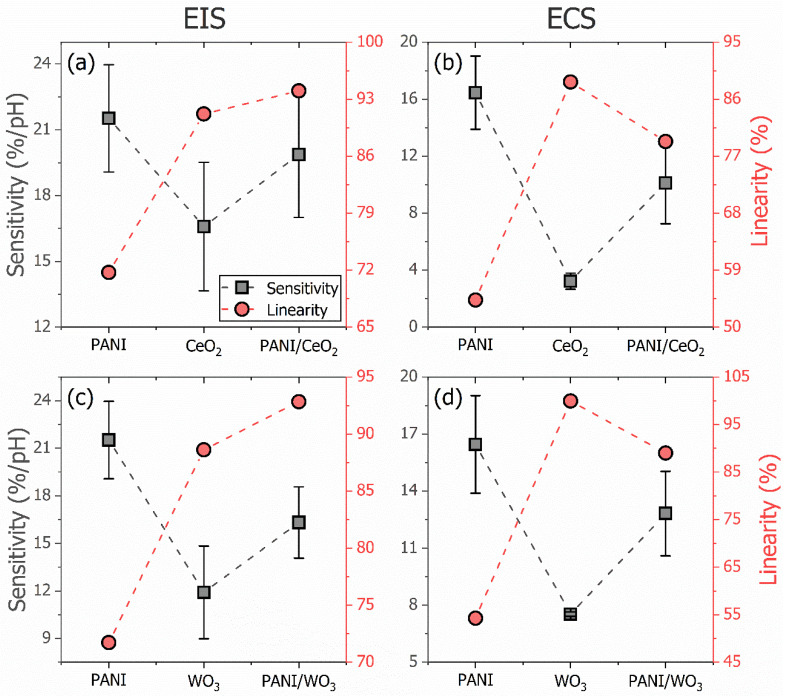
Comparison of the analytical values of merit (sensitivity and linearity) for the impedimetric (EIS) and capacitive (ECS) electrochemical sensors for each group of materials presented in this work. In (**a**,**b**), the EIS and ECS responses, respectively, of the CeO_2_ group of films, and in (**c**,**d**), for the WO_3_ group of films.

The sensitivity and linearity of the EIS and ECS electrochemical sensors for each group of materials are presented in Figure 6. Figure 6a shows the sensitivity and linearity of the impedimetric platform for each film in the CeO_2_ group. Figure 6b shows the capacitive results for the same set of films. The EIS sensitivity and linearity are 21.5 ± 2.4%/pH and 71.7%, respectively, for the PANI. For the CeO_2_, they are 16.6 ± 2.9%/pH and 91.2%, respectively. The sensor with CeO_2_ presents a lower sensitivity than the sensor with PANI. By contrast, the linearity is higher for the sensor with CeO_2_ than for that with PANI. The PANI/CeO_2_-based sensor presents a sensitivity of 19.9 ± 2.9%/pH and a linearity of *c.a.* 94%. Comparing the impedimetric (EIS) pH sensor with the spin-coated PANI thin film with the electrodeposited one from our previous work [39], the device from this work presents a higher sensitivity (21.5 ± 2.4%/pH vs. 12.7 ± 2.2%/pH). Regarding the linearity, it is lower for the spin-coated sample (71.7% vs. 91.7%). This is also observed for the capacitive (ECS) sensor.

The nanocomposite presents a synergic performance. The described results indicate that the nanocomposite’s sensitivity is in the level of the PANI’s sensitivity, and the linearity is in the level of the CeO_2_ oxide. The nanocomposite improves the polymer linearity and metal oxide sensitivity. The same behavior as for the EIS sensitivity is observed for the ECS sensor. The sensitivity is 16.5 ± 2.6%/pH for the PANI, 3.22 ± 0.6%/pH for the CeO_2_, and 10.1 ± 2.9%/pH for the PANI/CeO_2_. Although the ECS sensitivity of the nanocomposite is not at the level of the PANI sensitivity, it represents a 213% increase compared to the metal oxide sensitivity. At the same time, the impedimetric sensitivity increases by about 20%. Regarding the ECS linearity, the PANI has 54.3%, the CeO_2_ has 88.7%, and the PANI/CeO_2_ composite has 79.3%. As for the capacitive sensitivity, the linearity does not reach the level of the metal oxide, although it has a 46% increase when compared to the polymer linearity, while for the impedimetric linearity, the increase for the nanocomposite is about 31%. One unexpected finding is the extent to which the PANI/CeO_2_ nanocomposite optimizes the performance of the pH sensor for both impedimetric and capacitive platforms. This improves not only the linearity of the PANI-based sensor but also the sensitivity of the CeO_2_-based sensor.

Figure 6c,d present the sensitivity and linearity of the impedimetric and capacitive platforms, respectively, for each film in the WO_3_ group. The EIS sensitivity and linearity for the PANI are the same as for the previous group. For the WO_3_, they are 11.9 ± 2.9%/pH and 88.6%, respectively. Again, the metal oxide-based sensor presents a lower sensitivity and higher linearity than the polymeric sensor. The PANI/WO_3_ nanocomposite presents impedimetric sensitivity and linearity of 16.3 ± 2.2%/pH and 92.8%, respectively, improving on the polymer’s linearity and the metal oxide’s sensitivity. Although the nanocomposite’s sensitivity is not at the PANI’s sensitivity level, it represents an increase of 37% in relation to the WO_3_’s sensitivity. Regarding the linearity, the composite is higher than the metal oxide, with a rise of 5%, representing an increase of 31% concerning the PANI’s linearity. The ECS platform with the PANI/WO_3_ nanocomposite presents the same behavior as the platform with the PANI/CeO_2_. The nanocomposite presents a sensitivity improvement in relation to the metal oxide of 71%, from 7.5 ± 0.1%/pH to 12.8 ± 2.2%/pH, and a linearity improvement concerning the PANI of 64%, from 54.3% to 89%. Compared to previous work, it is possible to control the response of each sensor though the protonation potential and electronic density of the PANI sample by combining it with a metal oxide instead of controlling the oxidation state of the polymer.

The thin film morphology has a significant influence on the response of electrochemical interfacial sensors. The thickness and surface roughness, $R_{Q}$ (root mean square deviation), of the thin films are presented in Table 1. For each group of films, the CeO_2_ and WO_3_ films are the thickest (11.6 ± 1.8 and 21.1 ± 1.9 nm, respectively) and the composites, PANI/CeO_2_ and PANI/WO_3_, are thinner than the oxide. The PANI/CeO_2_ are thinner than the PANI film (4.2 ± 0.2 vs. 5.0 ± 1.0 nm), although the PANI/WO_3_ is thicker (11.7 ± 0.2 vs. 5.0 ± 1.0 nm). The interaction between the polymeric chain and the metal oxide generates a composite sample with unique structural properties.

**Figure 7:**
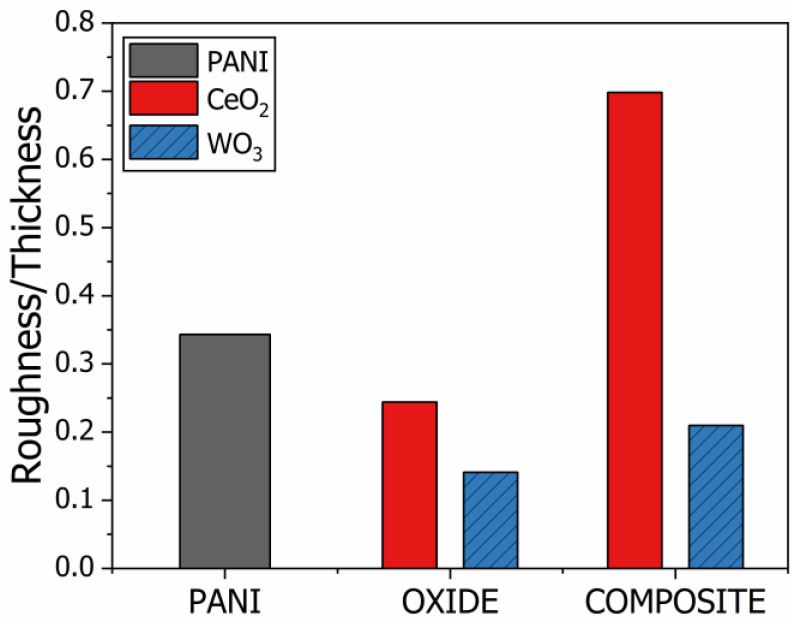
Analysis of the roughness-to-thickness ratio parameter for both sets of films. The red filled columns show the results for the CeO_2_ group of films, while the patterned blue filled columns show the results for the WO_3_ group of films.

Both the thickness and surface roughness are important for the interfacial processes at the film/electrolyte interface. While thicker films present a reduced electron transfer rate [61], electrodes with a higher surface area, directly related to the surface roughness, deliver an enlarged electron transfer rate [62]. As shown in Table 1, both parameters are related, with the thicker film presenting higher surface roughness and vice versa. Based on this, the roughness-to-surface ratio can be a helpful parameter for evaluating the quality of a sample when applied in electrochemical sensors. Figure 7 displays the roughness/thickness parameter for both sets of films. Based on the effects of the roughness and thickness on the interfacial phenomenon, lower ratios indicate reduced sensor sensitivity and performance, and vice-versa.

A nanocomposite is a material that combines its constituent properties. The consequences are noted in its new and synergic properties. The interaction between the metal oxide and the polymer is responsible for altering the structural properties of the thin films, such as the thickness and roughness, as well as the response of the electrochemical sensors based on the material. Ultimately, the sensors’ sensitivity is defined by the nanocomposite’s thickness and roughness. As shown in Figure 7, the relationship between the roughness/thickness ratio and the material is the same as that shown for the sensitivity (Figure 6). The synthesis of new composites may be key to the development of materials with tailored properties presenting the optimal performance in sensor devices. For example, the described polymeric composites with CeO_2_ and WO_3_ metal oxides could offer improved and optimized sensitivity and linearity compared to the constituent materials.

## 4. Conclusions

The present study was designed to determine the effect of nanocomposites of PANI with metal oxides on the response of electrochemical impedimetric and capacitive pH sensors. The thin films were produced via spin coating on FTO substrates from an NMP solution of the nanocomposites. The solutions were prepared by physically mixing the hydrothermally synthesized metal oxides, CeO_2_ and WO_3_, and PANI. This study has shown that the PANI/CeO_2_ and PANI/WO_3_ nanocomposites optimized the EIS- and ECS-based pH sensors. The ECS sensitivity of the PANI/CeO_2_ sample represented an increase of 213% compared to the metal oxide’s sensitivity, while for the EIS sensitivity, the increase was 20%. The linearity showed an increase of 46% compared to the PANI’s linearity based on the ECS method and 31% based on the EIS method. For the PANI/WO_3_ sample, the increase in the sensitivity of the EIS and ECS methods was 37% and 71%, respectively, while the increase in the linearity was 31% and 64%, respectively. The results of this study indicate that the nanocomposites improved not only the linearity of the PANI-based sensor but also the sensitivity of the metal oxide-based sensor. These findings were supported by the analysis of the roughness-to-thickness ratio, two morphological characteristics of the thin films with an impact on the interfacial electron transfer rate and, consequently, the sensors’ performance.

Although there is a need for further studies to evaluate the structural characteristics of the composites and their effect on sensing devices, the findings of this study contribute to our understanding of PANI and PANI-based nanocomposites applied to impedimetric and capacitive electrochemical sensors for ions. A key strength of the present study was the evaluation of the capacitive properties of PANI-based materials applied in chemical sensors following the novel findings of previous works.

## Figures and Tables

**Table 1:** The thickness and surface roughness, $R_{Q}$ (root mean square deviation), of the thin films from each group of materials.

Sample	Thickness (nm)	R_Q_ (nm)
PANI	5.0 ± 1.0	1.7
CeO_2_	11.6 ± 1.8	2.8
PANI/CeO_2_	4.2 ± 0.2	2.9
WO_3_	21.1 ± 1.9	3.0
PANI/WO_3_	11.7 ± 0.2	2.5

## Data Availability

The data presented in this study are available on request from the corresponding author.

## Supplementary Materials

The following supporting information can be downloaded at https://www.mdpi.com/article/10.3390/polym15030578/s1, Figure S1: CV of the metal oxide thin films, Figure S2: Absorption UV-VIS spectra of the PANI-EB and PANI-ES, Figure S3. UV-VIS spectra of the PANI-EB and PANI-ES thin films.
