# Supplementary material for: Thin Films of Polyaniline-Based Nanocomposites with CeO2 and WO3 Metal Oxides Applied to the Impedimetric and Capacitive Transducer Stages in Chemical Sensors
Source: Polymers (Basel). 2023 Jan 22;15(3):578. doi: 10.3390/polym15030578 (PMC9920537; doi:10.3390/polym15030578)
Supplement: Supplementary file 1 [file polymers-15-00578-s001.zip › polymers-2120502-supplementary.pdf]

Supporting Information

Thin films of polyaniline-based nanocomposites with CeO_2 and WO_3 metal oxides applied in impedimetric and capacitive transducer stages for chemical sensors

Beatriz Cotting Rossignatti[a], Amanda Portes Vieira[a], Martin Schwellberger Barbosa[b], Luís Miguel Gomes Abegão[a], Hugo José Nogueira Pedroza Dias Mello[a,*]

[a] Instituto de Física, Universidade Federal de Goiás, Campus Samambaia, 74001-970, Goiânia, GO, Brazil

[b] Instituto de Química, Universidade Federal de Goiás, Campus Samambaia, 74001-970, Goiânia, GO, Brazil

*Corresponding author: hugomello@ufg.br

1. Energy-dispersive X-ray spectroscopy of the nanocomposite thin films

Due to the sub 20 nm thickness of both composite films (Table 1, main text), the most intense EDS peaks can be attributed to signal that originated from elements present in the glass substrate (O, Na, Mg, Ca, Si). To avoid overlapping, Ce was identified by the peak correspondent to the Lα energy at 4.845 keV and W was identified by the peak correspondent to the Lα energy at 8.395 keV.

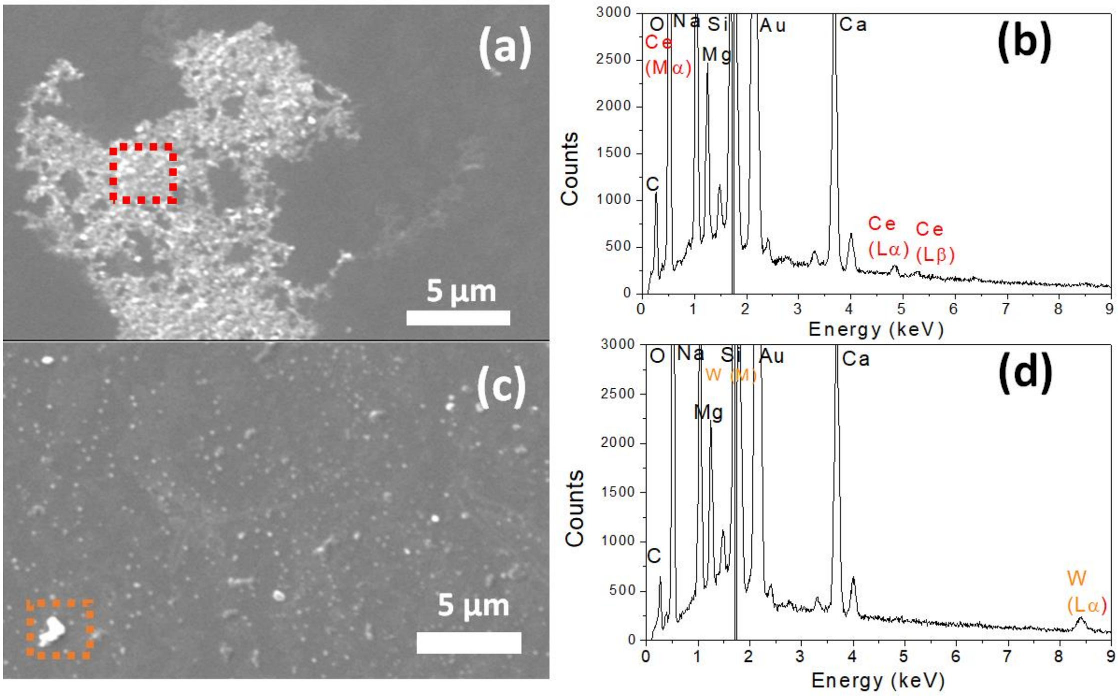

Figure S1. SEM-EDS characterization of the PANI/CeO2 (a) and (b) and PANI/WO3 films (c) and (d), deposited over glass substrates. The selected areas from which the EDS signal was collected are indicated by dashed lines.

2. Cyclic voltammograms of the metal oxides thin films

CeO_2 and WO_3 thin films deposited by spin-coating from a 60 and 11 mg/mL dispersion, in NMP, respectively, were characterized by cyclic voltammetry (CV) in a conventional three-electrode system. CeO_2 and WO_3 thin films were the working electrode, the reference electrode was an Ag|AgCl electrode, and a platinum foil was used as the counter electrode. CV was applied with a scan rate of 100 mV/s from – 0.2 to 1.2 V (vs Ag|AgCl) in each pH buffer solution.

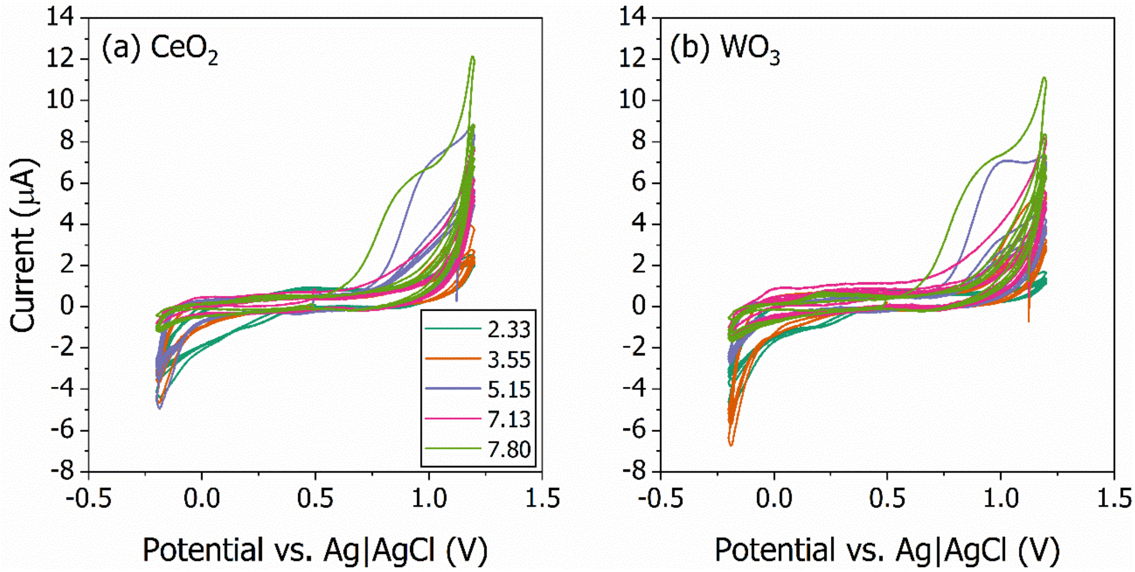

Figure S2. Cyclic voltammetry of the pH sensors for (a) CeO_2 and (b) WO_3. The samples did not show reversible redox peaks.

3. UV-VIS spectroscopy of doped and un-doped PANI thin films

PANI (PANI-EB) thin films were spin-coated (1000 rpm, for 60 s) using a spin-coater (G3P-8 SCS) from a weight ratio of 1:100 polymer:solvent (PANI:DMF, N,N-Dimethylformamide, 99 %, Sigma-Aldrich) solution. Glass thin films were used as substrate after cleaning. The samples were annealed on a hot plate for 20 min at 120 ° C. After deposited, a PANI sample was dipped in buffer solution with pH 2.2 for 30 min. This was the doped PANI sample (PANI-ES, emeraldine salt). The undoped sample is the PANI-EB (emeraldine base). Film thickness were obtained using a DektakXT stylus (Bruker) profilometer. The UV-VIS optical spectra of the samples were recorded using a Lambda 1050 WB (PerkinElmer) spectrophotometer in absorbance mode, from 370 to 850 nm in a 0.5 nm step. It is found that protonated PANI has a unique shoulder around 440 nm in the absorbance spectra.

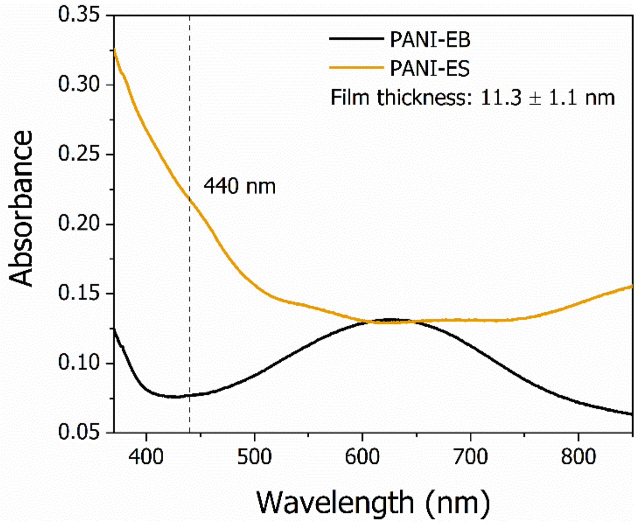

Figure S3. UV-VIS spectra of the PANI-EB and PANI-ES thin films. The dashed line indicate the shoulder near 440 nm which is uniquely related to the PANI-ES form [1].

References

1. Tarver, J.; Yoo, J.E.; Dennes, T.J.; Schwartz, J.; Loo, Y.-L. Polymer Acid Doped Polyaniline Is Electrochemically Stable Beyond PH 9. *Chem. Mater.* **2009**, *21*, 280–286, doi:10.1021/cm802314h.
